# Simplified Virtual Reality System Can Be Used to Evaluate the Temporal Discrimination Ability in Softball Batting as in the Real Environment

**DOI:** 10.3389/fspor.2022.843896

**Published:** 2022-04-25

**Authors:** Daiki Nasu, Takamichi Baba, Takumi Imamura, Masumi Yamaguchi, Yoshitake Kitanishi, Makio Kashino

**Affiliations:** ^1^NTT Communication Science Laboratories, NTT Corporation, Kanagawa, Japan; ^2^Biostatistics Center, Shionogi & Co., Ltd., Osaka, Japan; ^3^Department of Data Science, Shionogi & Co., Ltd., Osaka, Japan

**Keywords:** virtual reality, head-mounted display, movement onset time, cognitive-motor, hitting, marker-less pose estimation

## Abstract

Recently, virtual reality (VR) technology has developed rapidly and has increasingly come to be used in the sports field. VR technology ranges from large, highly immersive devices to simple devices such as smartphones, and the respective usefulness and shortcomings of different device types have been debated. Simple devices have advantages such as portability, but also provide only a weak sense of realism. It is important to understand the purpose and extent to which VR technologies can be used. Our purpose in this study was to briefly measure one of the cognitive-motor abilities used in softball batting: temporal discrimination ability in swing onset when a batter faces two types of balls thrown at different speeds. We investigated whether a simplified head-mounted display (HMD) system can evaluate such cognitive-motor ability to the same extent as in a real environment. Ten elite female softball batters swung at fastballs and slowballs randomly thrown by the same pitcher in both real and 3D VR environments, with the same range of trajectories. We then compared the temporal discrimination ability of swing onset analyzed by video analysis between environments. We found that the discrimination ability in VR is almost the same as in reality. In addition, questionnaire items on the VR system related to user experience and cybersickness showed overall promising responses. However, we also found that the system had some issues that need to be considered, such as leading to early swing onset and large variability in it. We discussed the usefulness and limitations of the VR system by combining the results for swing onset with the questionnaire responses. By understanding the characteristics of VR technology and using it as an efficient evaluation and training of players, the sports field can make significant progress.

## Introduction

Virtual reality (VR) technology is increasingly being used in the sports field, not only for entertainment but also for evaluating a player's ability and training (Michalski et al., 2019a). However, some players and coaches may be skeptical about the benefits of VR technology. Problems that have been pointed out involve hardware and software limitations of the current technology, such as the complexity of stereoscopic depth, cybersickness, and high cost (Miles et al., 2012). Even though it is difficult to reproduce realism perfectly, it is crucial to know the range of use depending on the purpose, environments, and sports events. In this study, we focused on the timing adjustment of softball batting. We examined whether our simplified VR system can be used to assess one of the cognitive-motor functions of players, by comparing it to a real situation.

In baseball/softball, a pitcher throws balls of various speeds and pitch types to overcome an opponent batter. Therefore, the batter needs to predict the spatial and temporal trajectories of the thrown ball and adjust his/her swing accordingly. In other words, batting requires a series of cognitive-motor processes to reflect the discrimination of pitch speed and type into the swinging motion in a short period of time. Many studies have focused on the temporal accuracy of swinging motion and highlighted its importance (Gray, 2002; Katsumata, 2007; Ranganathan and Carlton, 2007; Cañal-Bruland et al., 2015; Kidokoro et al., 2019, 2020; Takamido et al., 2019; Nasu et al., 2020). In particular, swing onset is considered to reflect the batter's initial decision regarding motor execution; the difference in swing onset time when hitting balls thrown at different speeds is a critical factor related to real game performance (Nasu et al., 2020). Therefore, measuring the temporal discrimination ability of swing onset is useful for evaluating a batter's cognitive-motor functions. However, to perform such measurements, it is necessary to prepare the measurement equipment, location, and time, which places a heavy burden on the opposing pitcher in particular. VR may solve these issues and make measurements easily, quickly, and at any location.

VR is defined as the use of computer modeling and simulation that enables a person to interact with an artificial 3D visual or other sensory environment (Lowood, 2021). Typical VR devices include PC monitors, data projectors often with large display walls, head-mounted displays (HMDs), and cave automatic virtual environments (CAVEs). VR technology can provide learners with a sense of realism and immersion, and it is expected to be more effective in sports training than traditional video training and other methods (Michalski et al., 2019a). Some studies have been conducted on the reliability of sports VR. For example, it has been reported that players' actions in VR environments can reproduce those in real environments for handball keepers (Bideau et al., 2003) and baseball batting (Isogawa et al., 2018). There are also reports that VR training improves sports performance in tennis (Jiang and Rekimoto, 2020), table tennis (Michalski et al., 2019b; Oagaz et al., 2021), and even leads to improved performance in a real game of baseball batting (Gray, 2017).

In this study, we investigated the temporal discrimination ability of swing onset when the softball batter faced two types of balls thrown at different speeds in both VR and real environments. We then examined whether our VR system can evaluate such cognitive-motor abilities to the same extent as in reality. We used a simple wireless HMD. The advantages of HMDs are their portability, low cost, and ease of programming. Players and coaches can easily use HMDs in the field at any time and place. Despite these advantages, it has also been reported that the HMD has some issues, such as reduced movement speed and stability (Pastel et al., 2020; Almajid et al., 2021; Chen et al., 2021), distance underestimation (Renner et al., 2013; Gerschütz et al., 2019), and cybersickness (Rebenitsch and Owen, 2016; Kourtesis et al., 2019) caused by technical limitations such as the narrow field of view (FOV), the weight of the device, and the lack of visibility of one's own body. To determine the existence of these issues, we also conducted a questionnaire investigation about the user experience and the cybersickness of the VR system. By combining the results for swing onsets with the subjective questionnaire feedback from the participants, we gained insight into the usefulness and limitations of the VR tool.

## Methods

### Participants

For comparison of swinging motion between the VR and real environments, 10 elite female softball batters participated in the experiments. They were competitive fast-pitch softball players from the Japan Softball Top League. The mean ± SD age was 24.4 ± 2.8 years, and height was 163.6 ± 5.2 cm. The number of years that they had played softball and/or baseball was 14.7 ± 3.5 years. Four of them were right-handed batters, and all of them were fielders (none were pitchers).

For questionnaire investigation, 16 elite female softball batters, including those who participated in the swinging experiments, participated. The age was 23.9 ± 3.5 years and the number of years that they had played softball and/or baseball was 15.6 ± 3.8 years.

All the participants provided written informed consent before participating in the experiments. This study was approved by the Shionogi Ethics Committee, in accordance with the Declaration of Helsinki.

### VR System

Participants wore a wireless HMD, Oculus Quest 2 (Facebook Technologies LLC, Menlo Park, CA, USA), which has a singular fast-switch LCD panel with a 1,832 × 1,920 per eye resolution and a weight of 503 g. The FOV was 89° in the horizontal direction and 93° in the vertical direction, and the maximum refresh rate was 90 Hz. Participants with prescription eyeglasses wore them while using the HMD.

To render the pitchers and ball trajectories in VR, we measured the pitching data of two female pitchers (pitchers A and B) from the same team as the participating batters before the experiments. Pitcher A was right-handed, and pitcher B was left-handed. Each pitcher threw 30 fastballs and 30 slowballs (change-up) to various locations in the strike zone. Their pitching motions were recorded by placing a video camera set at 60 fps with a 1,920 × 1,080 resolution (Sports Coaching Cam, JVCKENWOOD Corp., Yokohama, Japan) behind the catcher, and the physical parameters of each ball trajectory were also measured using Rapsodo 2.0 (Rapsodo LLC, Yokohama, Japan); that is, we prepared 60 datasets per pitcher. The ball speeds of pitcher A were 95.1 ± 0.9 km/h for the fastball and 68.5 ± 2.0 km/h for the slowball, and those of pitcher B were 92.5 ± 1.4 km/h for the fastball and 73.4 ± 1.7 km/h for the slowball.

**Figure 1:**
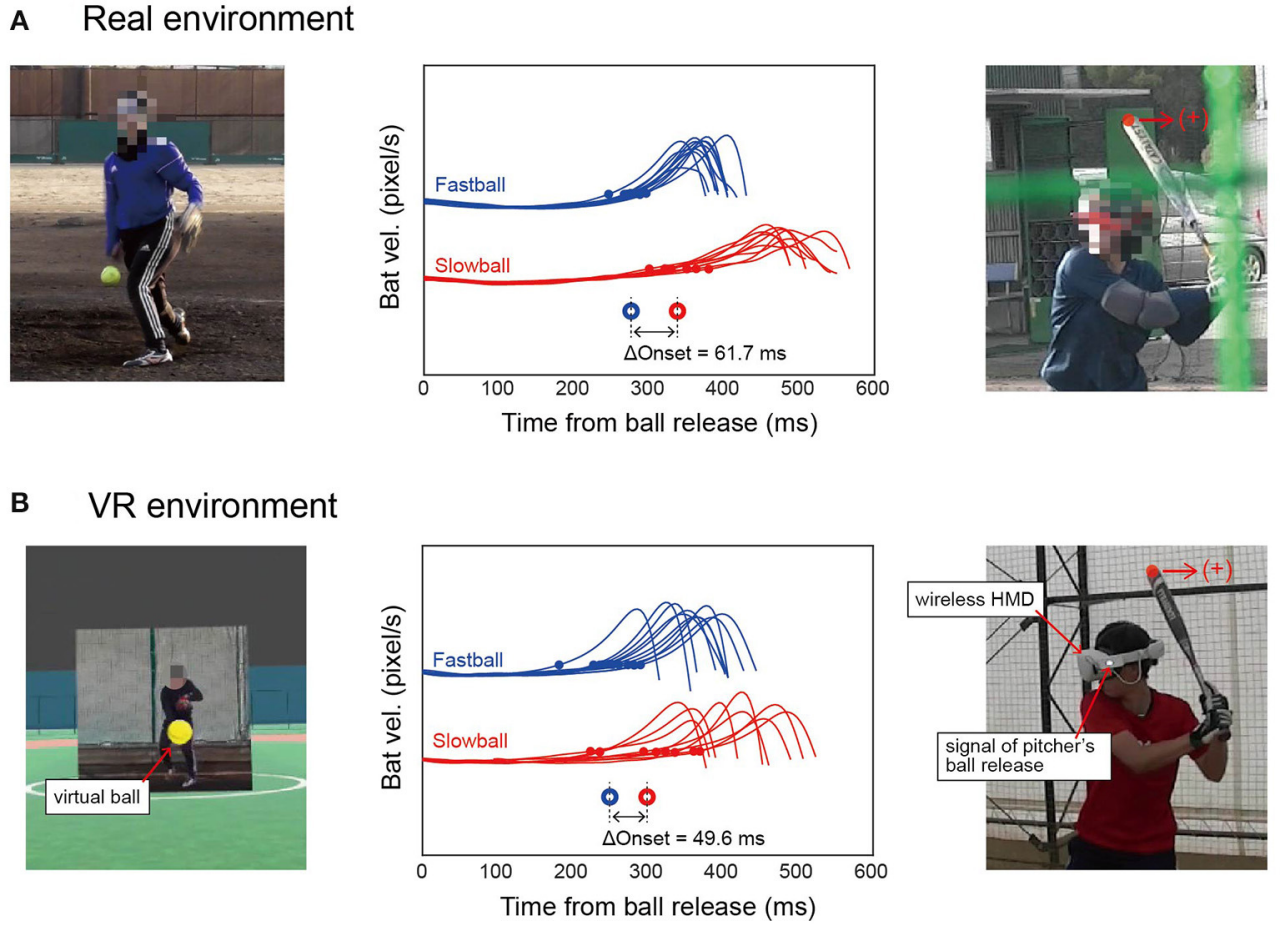
Experimental environments and time curves of bat velocities. **(A)** In the real environment, a batter hit balls thrown by a real pitcher. The right panel is a video image for identifying the batter's swing onset with the tip of the bat labeled (red dot). The center panel shows the time curves of the bat velocities in the catcher's direction, where the small dots are the swing onset and the large open circles are their average for each type of pitch. **(B)** In the VR environment, a batter swung at the balls thrown by a virtual pitcher. The left panel is a pitcher as seen by a batter in the VR. In the right panel, the pitcher's ball release was identified by the LED signal. The notation is the same as in **(A)**.

We constructed a softball field in VR. Then, we rendered the 2D video images of the pitcher at the position of the pitcher rubbers; balls appearing in the video were removed to avoid duplicate representations, referring to a previous study (Isogawa et al., 2018) (left panel of Figure 1B). The 3D ball trajectories were created by approximating them as a quadratic time function based on the actual ball trajectories obtained by Rapsodo 2.0. It should be noted that our VR system did not allow interaction with objects in the virtual world, as the system did not track the movement of the batter and bat online. This means that the batter could not hit the virtual balls even if they swung at them.

### Task and Apparatus

#### Real Environment

For the real environment comparison, the experiment was conducted in an outdoor field. Batters faced one of the two pitchers. After a sufficient warm-up, the batters hit two fastballs and two slowballs as practice. Then, the batters tried to hit the fastballs and slowballs thrown randomly by the pitcher. Each batter swung ten times at each pitch, but the pitch type was not announced in advance. Batters were instructed to swing only when the pitched ball was in the strike zone.

The pitcher's motion was captured using a video camera placed 10 m away from the pitcher to identify the ball release, and the batter's motion was also captured by another camera placed 5 m away from the batter. Both cameras (Sports Coaching Cam, JVCKENWOOD Corp., Yokohama, Japan) were set at 240 fps with a 640 × 480 resolution and synchronized by LED signals installed in front of each camera.

#### VR Environment

The experiment in the VR environment was conducted in an indoor field large enough to swing a bat. The order of tasks in the two environments was counterbalanced across the batters. Each batter wore the HMD and faced the same pitcher as in the real environment. After sufficient warm-up and practice hits (just as in the real environment), the batter swung against ten fastballs and ten slowballs thrown randomly by the pitcher in the VR environment. Again, batters could not hit the virtual balls in our system and get visual or tactile feedback. The pairs of videos of the pitching motion and 3D ball trajectories were randomly selected from the 60 datasets for each pitcher.

With our wireless VR system, we could not identify the pitcher's ball release in the same way as in the real environment, because the experimenters could not observe the virtual pitcher from the outside. We solved this problem by playing a sound at the time of the pitcher's ball release and converting the audio signal from the earphone jack into an LED signal (right panel of Figure 1B). The LED signal and batter's motion were captured by a video camera that was placed 5 m away from the batter and set at 240 fps with a 640 × 480 resolution.

### Questionnaire

Kourtesis et al. (2019) designed the Virtual Reality Neuroscience Questionnaire as a brief tool to appraise and report both the quality of software features and the intensity of VR induced symptoms and effects (i.e., cybersickness). Based on their questionnaire items related to user experience and cybersickness, we developed a questionnaire with 15 questions that included items related to HMD limitations (FOV and fitting of the headset) and softball batting (Table 1). The responses to each question were rated on a Likert scale of 1 to 7 (1: most negative, 7: most positive). We also asked the participants who gave negative responses (1 to 3) to each comment to write down their reasons. The participants who did not take part in the swinging experiments answered the questionnaire after experiencing the same procedure of the VR experiment as those who did.

**Table 1:** Questionnaire on the VR experience (*N* = 16).

		**Statement (1: most negative / 7: most positive)**	**Mean ± SD**	***n* (%)**
User experience	Q1	What is the level of immersion you experienced? (low / high)	4.2 ± 1.4	12 (75%)
	Q2	What was your level of enjoyment of the VR experience? (low / high)	4.9 ± 0.9	16 (100%)
	Q3	How was the quality of the graphics? (low / high)	4.8 ± 1.4	14 (88%)
	Q4	Do you think it is more effective to practice compared to viewing videos on a typical 2D monitor, etc.? (low / high)	5.1 ± 0.8	16 (100%)
	Q5	How did you feel about the field of view (FOV)? (narrow / wide)	4.6 ± 1.2	13 (81%)
	Q6	How did you feel about the comfort of the headset? (bad / good)	3.3 ± 1.2	4 (25%)
Cyber-sickness	Q7	Did you experience nausea? (feel / absent)	5.2 ± 1.7	13 (81%)
	Q8	Did you experience disorientation? (feel / absent)	4.9 ± 2.0	13 (81%)
	Q9	Did you experience dizziness? (feel / absent)	5.3 ± 1.7	13 (81%)
	Q10	Did you experience fatigue? (feel / absent)	5.0 ± 1.8	12 (75%)
Batting	Q11	Were you able to swing as you would in real batting? (No / Yes)	3.6 ± 1.8	7 (44%)
	Q12	How did you see the pitcher compared to the real one? (not similar / similar)	4.4 ± 1.5	10 (63%)
	Q13	How did you see the ball compared to the real one? (not similar / similar)	4.4 ± 1.3	12 (75%)
	Q14	Do you want to use it for practice? (No / Yes)	5.0 ± 1.3	15 (94%)
	Q15	What is your overall impression of this VR system? (bad / good)	5.0 ± 1.1	15 (94%)

*n is the number of responders who answered 4 or higher, and N is the total number of participants who answered the questionnaire. Percentage shows the proportion of n/N*.

### Data Analysis

We analyzed the recorded videos of the pitcher and batter's motions using DeepLabCut (Mathis et al., 2018; Nath et al., 2019). DeepLabCut is an efficient method for marker-less pose estimation based on transfer learning with deep neural networks. This open-source package is easy for anyone to install and use, including freely labeling user-defined markers. We installed DeepLabCut (ver. 2.1.10.4) on a PC with a GPU (GEFORCE RTX 3090, Nvidia Corp, Santa Clara, CA, USA). We selected 60 frames × three trials per pitcher from videos and labeled the pitcher's wrist and ball in the real environment. We also selected 60 frames × three trials per batter and labeled the bat tip in both environments (right panels of Figures 1A,B). The network was trained for 100,000 iterations until the loss plateaued. Markers with a likelihood of less than 0.9 were excluded. The 2D positions of the obtained markers were smoothened using a zero-lag fourth-order Butterworth low-pass filter with a cut-off frequency of 20 Hz for pitchers and 10 Hz for batters. It should be noted that we did not need to calibrate for actual length conversion because we only focused on the temporal aspects in this study, which made measurement much more effortless.

We defined the pitcher's ball release in a real environment based on the temporal pattern of the distance between the pitcher's wrist and the ball. Ball release was defined as the moment at which the distance exceeded 20 pixels.

We defined the batter's swing onset in both environments based on the temporal pattern of bat tip velocity. The swing onset was defined as the moment in which the velocity toward a catcher's direction exceeded a certain threshold, which was 10 % of the mean peak velocity for each batter (295.8 ± 75.0 pixel/s) (small dots in the center panels of Figures 1A,B).

In the real environment, swing onset was defined as the duration between the pitcher's ball release, identified by video analysis, and the batter's swing onset. In contrast, in the VR environment, it was defined as the duration between the LED lighting on the HMD and the batter's swing onset. The swing onset for each batter was averaged for fastballs and slowballs (circles in the center panels of Figures 1A,B). Then, the difference in swing onset between pitch types was calculated as the delta onset.

Statistical analyses were performed using the Statistics and Machine Learning Toolbox in MATLAB 2017b (MathWorks Inc., Natick, MA, USA). Pearson's correlation coefficients between the real and VR environments and their confidence intervals were calculated for the delta onset, and the swing onsets for the fastball, and slowball. The delta onset and swing onsets for the fastball and slowball were compared between the two environments using a paired *t*-test. Intra-individual SDs of the swing onsets for the fastball and the slowball were calculated as the variabilities and compared using a paired *t*-test. Note that the delta onset did not have variability because it was calculated as the difference in the mean value of swing onsets between the two pitch types. For paired *t*-tests, 95% confidence intervals for the differences between the two environments were calculated, and the significance level was set at *p* < 0.05.

The questionnaire results were indicated as mean ± SD for each question, and also calculated the mean values for each three subcategories. In addition, the numbers of responders who answered 4 or higher (n) and their proportions to the total participants were shown.

## Results

**Figure 2:**
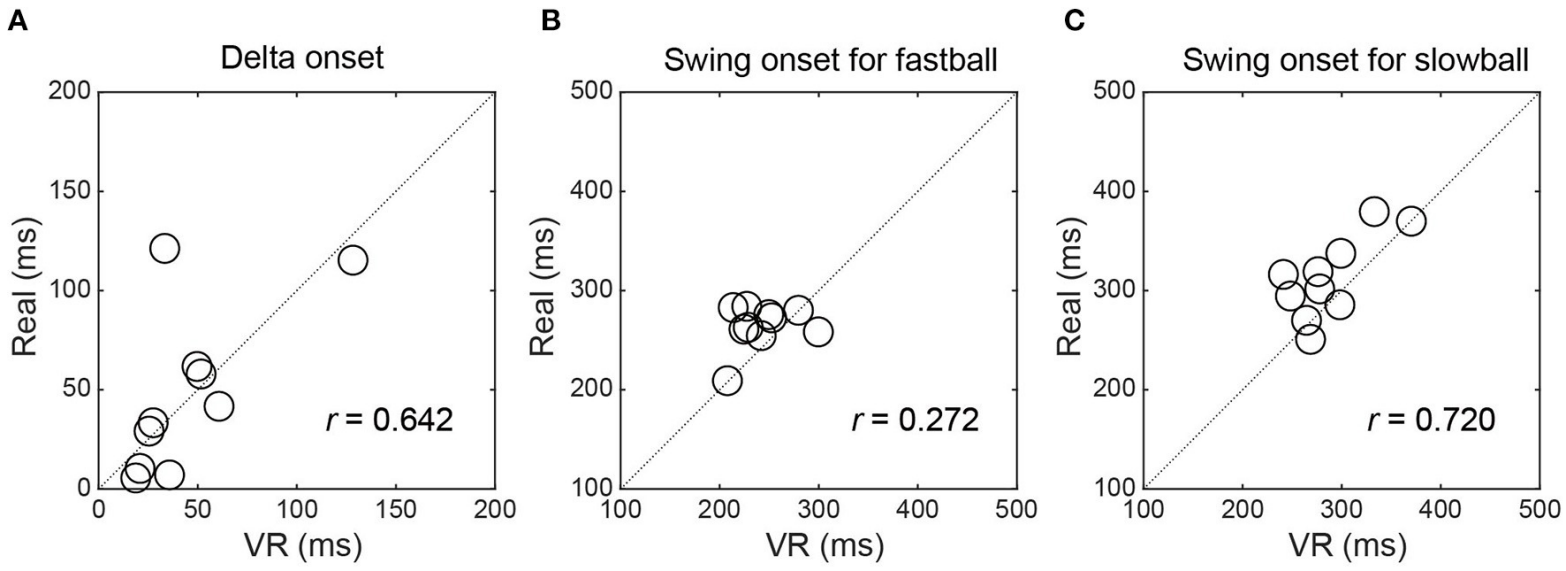
Relationships of the delta onset and swing onsets between real and VR environments. The delta onset **(A)** and the swing onset for the slowball **(C)** showed a high correlation between environments, while the swing onset for the fastball **(B)** showed a low correlation.

The delta onset showed a high positive correlation between the real and VR environments (*r* = 0.642, 95% CI [0.021 0.906]) (Figure 2A). The swing onset for slowball showed a high positive correlation between the two environments (*r* = 0.720, 95% CI [0.166 0.929]) (Figure 2C), while that for fastball showed a low correlation (*r* = 0.272, 95% CI [-0.431 0.770]) (Figure 2B).

**Figure 3:**
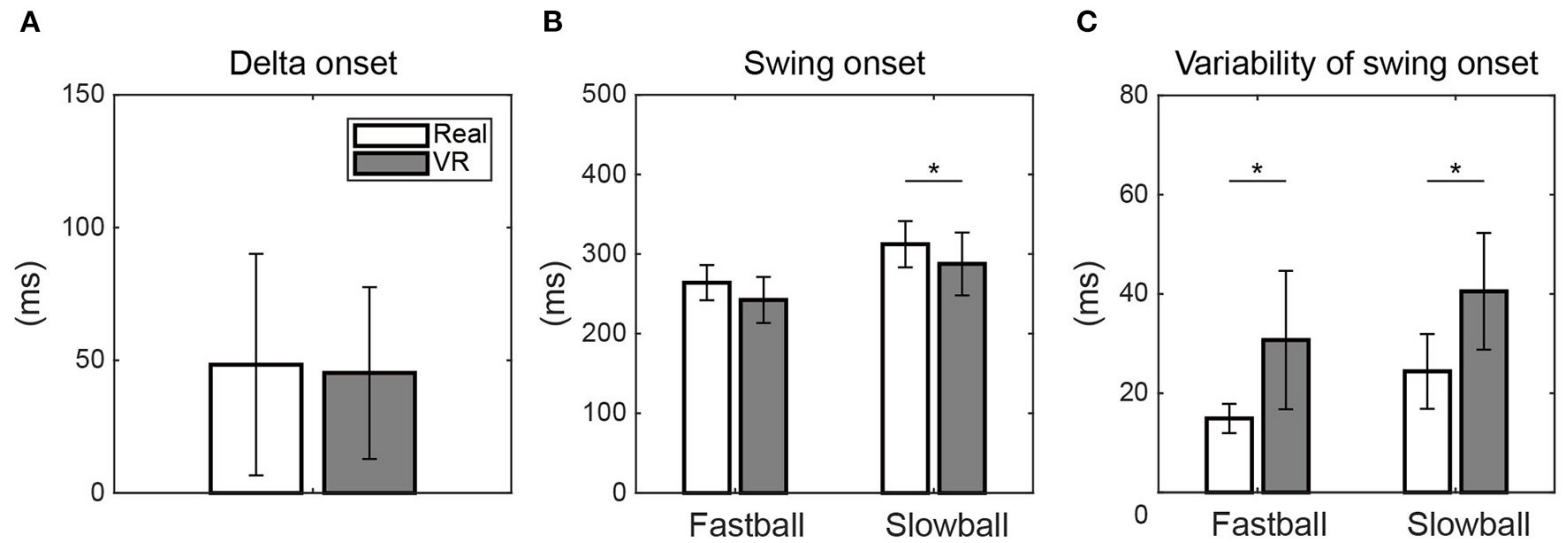
The delta onset, swing onsets, and variabilities of swing onset. **(A)** The delta onset was not significantly different between the real and the VR environments. **(B)** The swing onset for slowball was significantly earlier in the VR environment than in the real environment (*p < 0.05). **(C)** The intra-individual variabilities (SDs) of swing onset for both pitch types were significantly larger in the VR environment than in the real environment.

The delta onset was not significantly different between the real and VR environments {*t* (9) = 0.31, *p* = 0.766, 95% CI [-20.1 26.4]} (Figure 3A). The swing onset for fastball tended to be earlier in VR environment than in the real environment {*t* (9) = 2.19, *p* = 0.056, 95% CI [-0.73 44.0]}, and that for slowball was significantly earlier in the VR {*t* (9) = 2.60, *p* < 0.05, 95% CI [3.2 46.4]} (Figure 3B).

The variabilities of swing onset for both pitch types were significantly larger in the VR environment than in the real environment {fastball: *t* (9) = −3.37, *p* < 0.01, 95% CI [-26.4 −5.2], slowball: *t* (9) = −4.86, *p* < 0.01, 95% CI [-23.6 −8.6]} (Figure 3C).

The summary statistics of the questionnaire are shown in Table 1. Sixteen of the participants answered the questionnaire. Mean of responses related to user experience was 4.5, to cybersickness was 5.1, and to softball batting was 4.4. Overall, the responses to 13 of the 15 questions were positive scores (4.0 or higher), indicating a mostly satisfactory evaluation of the VR system. However, responses to two questions (Q6 and Q11) were negative scores (<4.0). Twelve and nine participants reported negative scores on Q6 and Q11, respectively. The reasons for the negative responses to Q6, wearing comfort of the VR headset, were: “it was heavy,” “shifted when swinging,” and “hurt my neck a little.” The reasons for the negative responses to Q11, the comparison with the real swing, were: “The direction of my head is different from usual due to the narrow FOV,” “It was difficult to swing as usual because of the lack of visibility of the motion of myself and the bat,” and “I feel uncomfortable due to the lack of hitting sensation.”

## Discussion

We investigated whether the delta onset could be assessed using the simplified VR system in the same way as in the real environment. The results showed that the VR system can evaluate the temporal discrimination ability to almost the same extent as in a real environment (Figures 2A, 3A). In addition, the results of the questionnaire also showed overall positive evaluations (Table 1). Nasu et al. (2020) conducted an experiment similar to the real environment experiment of this study to measure the delta onset. They found that the delta onset was related to the real game performance (i.e., the season batting average) and concluded that it was one of the crucial indicators to assess the batter's cognitive motor ability. Thus, the results of the current study indicate that coaches and analysts can use the VR system to assess the player's ability anytime and anywhere. For example, a quick assessment at regular intervals or before and after special training sessions could monitor the player's development or the effectiveness of the training.

Both late swing onset for the fastball and early swing onset for the slowball have the potential to reduce the delta onset. Our results showed a low correlation in the swing onset for fastball between the two environments because of small inter-individual variability (Figure 2B), but a high correlation for slowball because of the large variability (Figure 2C). In other words, the inter-individual variability of the delta onset seems to involve variability for the slowball and not for the fastball. Indeed, the delta onset was more correlated with the swing onset for slowball than for fastball in both environments (real: *r* = −0.290 for fastball, *r* = 0.859 for slowball, VR: *r* = −0.179 for fastball, *r* = 0.691 for slowball). It has been considered that baseball/softball batters expect and prepare a fastball when facing various ball speeds, and the inhibitory process of waiting for a slowball is a key factor (Gray, 2009; Cañal-Bruland et al., 2015; Muraskin et al., 2015; Nasu et al., 2020). The fact that we were able to analyze such key elements in the same way as the real environment also indicates the usefulness of this VR system.

While we focused only on the temporal component in batting, our VR might also be used for recognition of spatial location (i.e., identifying ball or strike). It has been widely reported that a 2D video-based system can assess not only the temporal aspect in batting but also spatial recognition ability (Müller et al., 2016). The VR-based sports training is considered to be more effective than 2D-based ones (Isogawa et al., 2018; Michalski et al., 2019a), and our participants also evaluated our VR system in the same manner (Q4 in Table 1). Whether HMD-VR devices are really effective in evaluating the spatial component as well as the temporal one requires future research, but using a VR system may allow us to more reliably assess such abilities for athletes.

On the other hand, we also found that the swing onsets in the VR have some differences from in the real environments: the swing onsets for both pitch types in VR were approximately 20 ms earlier (Figure 3B) and more variable than in real (center panels of Figures 1, 3C). These discrepancies may be caused by some technical limitations specific to HMDs. The first limitation is the narrow FOV. The FOV of our device was 93° in horizontal, which is less than half of actual human FOV, which can cover more than 190° (Howard and Rogers, 1996). The second is non-visualization of the user's body. There is a way to capture the participant's body movement and present his/her avatar in real time within the VR (Pastel et al., 2020), but this was not done in this study. The third is the weight of the HMD itself. Some of our participants pointed out their discomfort with the narrow FOV and the lack of visibility of their own motion. In particular, there were complaints about the device's weight, which scored negatively on average (Q6 in Table 1).

These limitations may lead to problems with human motion and visual perception. It has been reported that wearing an HMD may impair balance ability and reduce movement speed because of the additional weight of the device and restricted FOV (Almajid et al., 2021; Chen et al., 2021). The lack of visualization of one's own body also causes decrease in movement speed (Pastel et al., 2020). In this study as well, wearing the HMD might reduce the stability of the batter's movement and decrease their swing speed, making their swing onset earlier for proper timing of the bat-ball contact. Other studies have also reported underestimation of distances in VR due to the HMD's weight and limited FOV (Renner et al., 2013; Gerschütz et al., 2019) and the invisibility of one's own body (Naceri et al., 2009). Some of our participants also reported that “it was difficult to perceive the distance from the pitcher.” If a batter perceives the distance to the pitcher as closer than it actually is, this misperception may cause early swing onset. In the questionnaire, the mean response score to Q11, “Were you able to swing as you would in real batting?” showed a negative response. The participants provided their reasons as narrow FOV, weight of the device, and invisibility of the body and bat. Taken together, these technical limitations seemed to affect both the swing motion and the subjective perception.

There are other issues that need to be considered when using VR systems. Among them, the cybersickness is the one that needs special attention. “Cybersickness is the onset of nausea, oculomotor, and/or disorientation while experiencing virtual environments in head-mounted displays, large screens, and curved screen systems” (Rebenitsch and Owen, 2016). The presence of such symptoms is obviously undesirable. Particularly in sports VR, they may prevent the correct assessment of an athlete's abilities, weaken the training effect, and adversely affect the athlete's performance. The cause of cybersickness is commonly postulated to be a mismatch between the sensory input presented within the VR (e.g., visual input) and the sensory input from the external environment (e.g., vestibular input), or postural instability (Rebenitsch and Owen, 2016). However, the cybersickness seems to be technically being solved in the newer generation of HMDs, and the Oculus Quest2 used in this study generally meets the specs that are supposed to reduce the onset of cybersickness (Kourtesis et al., 2019; Kim et al., 2021). Indeed, in our questionnaire, the overall score for the questions about cybersickness was positive. However, there were a few participants who complained of sicknesses, so it is necessary to monitor the condition of participants while using the VR system.

Other issues that need to be taken into consideration include the fact that there are individual differences in adaptation to VR space. It has been reported that the feeling of being spatially located in the virtual environment differs between individuals and is related to differences in one's general spatial abilities (Coxon et al., 2016). More recently, it was also reported that there are individual differences in the rate of improvement through VR training in sports, and that these differences can be explained by differences in the structure of brain regions related to stereopsis and depth perception (Hosoda et al., 2021). In the current study, one of the ten batters showed a lower delta onset in VR than in the real environment (positioned at the upper left in Figure 2A). We cannot prove the reason for her result, but individual differences in visual properties related to spatial perception might have affected it.

As described above, our simplified VR has several limitations that may cause problems for certain purposes. For example, large variability of the swing onset in the VR would be an issue when investigating the delta onset between two pitch types with smaller differences in speed than we did. For such a purpose analyses should be interpreted after considering the large variability, increasing the number of trials, or using real environments or other advanced VR. For instance, using VR with a large screen on the wall like the CAVE, the head can be released from the pressure of the device, which would solve limitations such as narrow FOV, lack of body visualization, and HMD weight (Broll et al., 2022). There are also reports that distance perception was more accurate with large screen VR than with the HMD (Plumert et al., 2005; Naceri et al., 2009). Therefore, the use of screen-based VR may resolve the discrepancies between VR and real environments observed in this study.

There is, however, a trade-off between solving all the problems and (thereby) making the VR environment more realistic on the one hand, and saving time, space, and money on the other. For example, in our system, batters could not hit the ball because of lack of interactivity with the virtual world. It is considered that physical fidelity, such as feeling via visual and haptic rendering, must be replicated to maximize the effectiveness of VR (Michalski et al., 2019a). However, it may be costly to track the batter and bat motions and create a feeling of hitting the ball, as in a real environment. We concentrated on a simplified evaluation of one of the most important elements of softball batting (i.e., the delta onset) and adopted the HMD system that is portable, easy to use, and low cost, and proved its usefulness. It is important for coaches to learn about the detailed uses of the system that they plan to implement. Additionally, the coaches should understand the advantages and disadvantages of the system, and then determine whether or not the system fits their purpose.

The current study proved that the simplified VR system can evaluate a part of the cognitive-motor abilities used in softball batting as well as in the real environment. At the same time, we also discussed the several limitations that VR still has. VR technology, which is expected to continue to develop in the future, will drastically change the evaluating and training of athletes, and their overall sports entertainment. In the near future, the coaches and athletes who understand and utilize these pros and cons to efficiently enhance their abilities may survive.

## Data Availability Statement

The raw data supporting the conclusions of this article will be made available by the authors, without undue reservation.

## Ethics Statement

The studies involving human participants were reviewed and approved by the Shionogi Ethics Committee. The patients/participants provided their written informed consent to participate in this study.

## Author Contributions

DN, TB, TI, and YK designed and performed the experiments. DN built the VR system, analyzed the data, and drafted the manuscript. MY contributed to rendering the ball trajectories in the VR. DN, TB, TI, MY, and MK discussed the results and reviewed the manuscript. All authors approved the submitted version.

## Funding

This work was supported by a research grant from NTT for open access publication fees.

## Conflict of Interest

DN, MY, and MK are employees of NTT Communication Science Laboratories, which is a basic science research section of Nippon Telegraph and Telephone Corporation. TB, TI, and YK are employees of Shionogi & Co., Ltd. There is a pending patent in the literature.

## Publisher's Note

All claims expressed in this article are solely those of the authors and do not necessarily represent those of their affiliated organizations, or those of the publisher, the editors and the reviewers. Any product that may be evaluated in this article, or claim that may be made by its manufacturer, is not guaranteed or endorsed by the publisher.
